# Storage and purification adaptations for the isolation of total RNA from the dura mater

**DOI:** 10.1055/s-0042-1758865

**Published:** 2022-12-29

**Authors:** Maria Rosana de Souza Ferreira, André Pukey Oliveira Galvão, Marcelo Moraes Valença, Danyelly Bruneska Gondim Martins

**Affiliations:** 1 Laboratório de Imunopatologia Keizo Asami, Grupo de Prospecção Molecular e Bioinformática, Recife PE, Brazil.; 2 Universidade Federal de Pernambuco, Centro Acadêmico de Vitória, Departamento de Anatomia, Vitória de Santo Antão PE, Brazil.; 3 Centro Universitário FACOL, Vitória de Santo Antão PE, Brazil.; 4 Universidade Federal de Pernambuco, Unidade de Neurocirurgia, Departamento de Neuropsiquiatria, Recife PE, Brazil.; 5 Universidade Federal de Pernambuco, Departamento de Bioquímica, Recife PE, Brazil.

**Keywords:** Dura Mater, Nucleic Acids, Gene Expression, RNA, Dura-máter, Ácidos Nucleicos, Expressão Gênica, RNA.

## Abstract

**Background** RNA extraction is a step that precedes several molecular techniques. The fibrous tissue, more specifically the dura mater, has several limitations in routine protocols, and lacks optimization protocols to overcome these problems.

**Objective** To test stock reagents and purification kits, optimizing commercial kit protocols for RNA extraction from the dura mater.

**Methods** Dura mater samples were obtained from eight Wistar rats and maintained in two different stabilizers. The samples were purified using four different protocols, and the RNA was evaluated for the yield and purity in NanoDrop 2000 (Thermo Scientific, Wilmington, DE, United States). Beta-actin gene was used for analyzing gene expression, since is one of the most used reference genes.

**Results** The RNA preservation was similar in both stabilizers. The addition of an incubation step prior the purification protocols allowed better tissue digestion and RNA recovery. The RNA purified using the protocols membrane-based showed higher quality than liquid-liquid purification. This impact was observed in the 3-week evaluation using RT-qPCR.

**Conclusion** Stabilizers are efficient for RNA preservation and membrane-based purification protocols are more suitable for RNA recovery from dura mater tissue, allowing the evaluation of gene expression in this type of tissue. Adaptations in the dura mater RNA extraction protocol differ from the pre-established protocols because it takes into account the peculiarity of fibrous tissue and low cellularity. In addition to providing a low-cost mechanism, based on techniques that are part of the laboratory routine, it is possible to improve the quality of the extracted material, ensuring greater efficiency in the use of subsequent techniques.

## Resumo

**Antecedentes** A extração de RNA é uma etapa que antecede várias técnicas moleculares. O tecido fibroso, mais especificamente a dura-máter, apresenta várias limitações nos protocolos de rotina e carece de protocolos de otimização para superar estes problemas.

**Objetivo** Testar reagentes de estoque e kits de purificação, otimizando protocolos de kits comerciais para extração de RNA da dura-máter.

**Métodos** Amostras de dura-máter foram obtidas de oito ratos Wistar e mantidas em dois estabilizadores diferentes. As amostras foram purificadas em quatro protocolos diferentes e o RNA foi avaliado quanto ao rendimento e pureza no NanoDrop 2000 (Thermo Scientific, Wilmington, DE, United States). O gene da beta-actina foi utilizado para analisar a expressão gênica, uma vez que é um dos genes de referência mais utilizados.

**Resultados** A preservação do RNA foi semelhante em ambos os estabilizadores. A adição de uma etapa de incubação antes dos protocolos de purificação permitiu uma melhor digestão do tecido e recuperação de RNA. O RNA purificado pelos protocolos baseados em membrana apresentou qualidade superior ao da purificação líquido-líquido. Este impacto foi observado na avaliação de três semanas usando RT-qPCR.

**Conclusão** Os estabilizadores são eficientes para preservação do RNA e os protocolos de purificação baseados em membrana são mais adequados para recuperação de RNA do tecido da dura-máter, permitindo a avaliação da expressão gênica neste tipo de tecido. As adaptações no protocolo de extração de RNA da dura-máter diferem dos protocolos preestabelecidos porque leva em consideração a peculiaridade do tecido fibroso e com baixa celularidade. Além de fornecer um mecanismo de baixo custo, baseado em técnicas que fazem parte da rotina laboratorial, é possível melhorar a qualidade do material extraído, garantindo maior eficácia no uso de técnicas subsequentes.

## INTRODUCTION

The dura mater, the meninge that lines the central nervous system externally, is classified as fibrous, due to a large number of collagen fibers, making it a thick and resistant membrane. 1 The dura mater has two leaflets, an internal and an external leaflet, which have an embryological origin from the neural crest cells and the mesoderm, respectively. 2 The main functions of the dura mater are isolation, mechanical protection and stabilization of the central nervous system. In addition, the dura mater is richly vascularized and innervated, mainly by the branches of the trigeminal nerve. 3 As the brain has no sensitive nerve endings, all intracranial sensitivity is located in the dura and blood vessels, which appear to be involved in the pathophysiology of several forms of headache. 4 In addition to being involved in headache attacks, the blood vessels that are related to the dura mater are involved in different diseases and pathological manifestations. 3

However, in order to understand the pathophysiological mechanisms of some diseases, molecular analyzes are applied. To perform molecular techniques, it is necessary to extract from the tissue molecules, such as nucleic acids and proteins, which are employed in subsequent techniques. High-quality RNA is required for various techniques, including sequencing, reverse transcription quantitative polymerase chain reaction (RT-qPCR), microarray, etc. 5 Extraction and purification of RNA from fibrous tissues are challenging because it is a very rigid tissue with a low amount of nucleic acids and proteins. In addition, the number of cells in such tissue is generally very low, which leads to low RNA yield. 6

There are many commercial products available; however, most of the time RNA extraction kits do not bring specifications/adaptations to the different existing tissue types, often leading to a low-yielding RNA without precision and reliability of results. Not only in the case of fibrous tissues but some adaptations have already been made in other types of tissue, such as adipose tissue, since many protocols routinely used to isolate RNA from adipose tissue result in partially degraded RNA and/or low RNA yield or the small RNAs are lost in the process. 5 7 However, for peculiar types of tissue, there is a lack of information to guide the researcher in the preservation and purification protocols.

Therefore, the aim of the present article was to evaluate different combinations of commercially available kits for dura mater maintenance and RNA purification in order to establish a suitable combination between stock and purification reagent and evaluate the viability of RNA through the repeatability of gene expression.

## METHODS

### Animals

All experiments were developed according to the guidelines of the Animal Ethics Commission of the Universidade Federal de Pernambuco. Eight adult Wistar rats (between 8 and 10 weeks old) were purchased at the animal testing facility at the Academic Center of Vitória at the Universidade Federal de Pernambuco. Before surgeries to remove the dura mater, the animals were individually conditioned in polypropylene cages, stored in rooms under standardized laboratory conditions of temperature (22 ± 2°C), relative humidity (45 ± 5%), exhaust system with air renewal, luminance of 60 lux, and circadian cycle of 12 hours in light period and 12 hours in dark period. During the period of biological maintenance, the animals were fed with commercial feed and water *ad libitum* .

### Withdrawal of dura mater

The animals were anesthetized with ketamine (115 mg/kg) and xylazine (10 mg/kg), followed by tissue perfusion with 0.9% saline solution. To perform the dura mater collection procedure, the calvaria was removed through an incision in the occipitofrontal direction, the dura mater underlying the overlying bone disk was removed with the help of an 18-cm Molt detacher (Quinelato, Rio Claro, SP, Brazil). Two dura mater samples were collected from each animal, weighing between 5.0 and 8.6 ηg. The samples removed were quickly preserved in the following stabilization solutions: TRIzol (Thermo Fisher Scientific, Waltham, MA, USA; cat. number 15596026) or RNA *later* (Thermo Fisher Scientific, Waltham, MA, USA; cat. number AM7021). Then, samples were frozen at - 80°C for subsequent RNA extraction and purification.

### Dura mater RNA purification

RNA isolation was performed in duplicate using the following kits: TRIzol Reagent (Thermo Fisher Scientific, Waltham, MA, USA; cat. number 15596026); RNeasy Mini Kit (Qiagen, Hilden, Mettmann, Germany; cat. number 74104); Direct-zol (Zymo, Irvine, CA, United States; cat number R2070T), and ReliaPrep (Promega, Madison, WI, United States; cat. number: Z6110). The kits were tested for stored samples TRIzol and RNA *later* , according to the compatibility of each of them with solvents. The samples were quantified on the NanoDrop 2000 spectrophotometer (Thermo Scientific). The manufacturer protocol was followed, with minor modifications. A pretreatment was adopted for all kits, in which the samples were placed outside the freezer 24 hours before RNA extraction, being maintained at room temperature (19°C to 21°C) to assure complete thawing of all samples, and to allow the TRIzol reagent to act for sample digesting. Additionally, samples preserved in RNA *later* were incubated for 10 minutes at 60°C in a water bath before extraction to dilute the crystals in the solution. In the first step of separation in the purification protocol, all samples were submitted to a centrifugation time of 5 minutes at 12,000 × *g* , at 4°C.

### cDNA preparation and real-time PCR evaluation

For cDNA synthesis, the QuantiTect Reverse Transcription Kit (Qiagen, Hilden, Mettmann, Germany; cat number 205311) was used following its standard protocol, which is divided into two main steps: the removal of genomic DNA (gDNA) and the synthesis of cDNA. In the first step, 0.5 µl of the gDNA Wipeout Buffer was added to 3.0 µl of the total RNA. The first cycle was performed on the Thermocycler (Applied Biosystems, Waltham, Massachusetts, United States, 96-well thermal cycler, 0.2 ml, ref. 4375786), at a temperature of 42°C for 2 minutes, then the tubes were immediately placed on ice. For cDNA synthesis, 1.5 µl of a mix containing 0.25 µl of reverse transcriptase, 1 µl RT Buffer, and 0.25 RT µl primer mix was added to the sample. The samples went through the second cycle of 15 minutes at 42°C, 3 minutes at 95°C and 4°C ∞. Quantification was performed on the NanoDrop 2000 spectrophotometer (Thermo Scientific, Wilmington, DE, United States).

The qPCR reaction was performed by the StepOne Real-Time PCR-System (Applied Biosystems, Foster City, CA, USA) with the GoTaq qPCR Master Mix kit (Promega). Each PCR was performed in duplicates, in 96-well plates. They were used with fixed volumes, 5 μL SYBR (asymmetrical cyanine dye – SYBR Green I) and 1 μL of Carboxy-X-Rhodamine (CXR). A total of 0.2 μL of the beta-actin reference gene (Actb), Rn_Actb_1_SG assay (QuantiTect Primer Assay, Qiagen, Germany) was added. To complete the volume of 9 μL of reagents, 2.8 μL of water was added. Finally, 1μL of the cDNA sample was added, totaling the final reaction volume with 10 μL. For cycling, the fast protocol was used with 1 initial cycle of 95°C for 2 minutes for activation of Taq DNA polymerase, followed by 40 cycles of denaturation (95°C for 3 seconds) and annealing (60°C for 30 seconds). To assess the quality of the cDNA over the days, three qPCR plates were performed for 3 weeks. During the 3 weeks in which the plates were being performed, the cDNA samples were kept at −80°C.

### Data processing

The Ct values were expressed as mean and standard deviation (SD), calculated based on the values obtained over the 3 weeks. In the analysis of intrarepeatability, the mean and SD between duplicates were calculated over the 3 weeks.

## RESULTS

### Dura mater RNA purification

The present study aimed to optimize and evaluate yield and purity levels of dura mater total RNA isolation protocols based on commercially established methods. An additional step of thawing the sample 24 hours before the procedure facilitated the RNA purification once the phase separation after the centrifugation at the first steps of the extractions became more evident. Table 1 shows the quantitative measurements of the total isolated RNA, the mean, and SD of the Ct values over the 3 weeks. In all samples, the elution volume was the same, regardless the tissue mass.

**Table 1:** Quantitative measurements of the RNA purification, and RT-qPCR evaluation on average Ct over 3 weeks

Storage reagent	Purification kit	Sample	Tissue (ηg)	RNA yield (ηg/μL)	260/280	260/230	β-actin (ΔCt)	Intrarepeatability mean (ΔCt)
TRIzol	TRIzol	R1A	8.4	13.0	1.55	2.45	37.2 ± 15.2	36.7 ± 0.5*
		R1B	7.5	36.5	1.42	1.54	36.2 ± 18.7	
TRIzol	Direct-zol	R2A	7.6	4.3	1.57	- 0.04	26.2 ± 3.1	26.3 ± 0.1
		R2B	6.9	9.4	1.73	- 0.08	26.4 ± 3.1	
TRIzol	ReliaPrep	R3A	8.6	16.1	1.84	1.83	28.3 ± 3.2	28.8 ± 0.5
		R3B	6.3	7.2	1.80	2.00	29.4 ± 3.3	
TRIzol	RNeasy	R4A	6.3	5.5	1.69	0.35	30.1 ± 3.2	28.5 ± 1.6
		R4B	8.1	9.5	1.83	0.58	26.9 ± 2.8	
RNA *later*	ReliaPrep	R5A	5.9	5.0	1.89	- 6.36	26.2 ± 2.8	25.6 ± 0.6
		R5B	7.1	8.5	2.15	5.52	25.0 ± 2.9	
RNA *later*	RNeasy	R6A	5.0	19.2	2.00	1.21	27.2 ± 2.9	27.8 ± 0.6
		R6B	6.2	21.3	1.80	1.02	28.4 ± 3.2	

Notes: All samples were maintained at −80°C until the gene expression analysis.

*Samples showed expression only in the first of the three tests performed with β-actin.

The RNA yield could not be related to the sample weight, since high RNA yield could be obtained even in samples with lower mass input, varying from 5.5 to 35.5 ηg/μL. Regarding the RNA purity against DNA contamination, 50% of samples fit in the acceptable range (Abs 260/280 = 1.80 to 2.00). Only 1 sample was above the limit, and the others reaching values as low as 1.42. However, the deviation was significant for protein contamination, with most samples showing very low or even negative records in some samples. Only one sample fit in the range (Abs 260/230 = 2.0–2.2). The TRIzol/TRIzol combination was tested twice (in duplicate), but only the second experiment was included in the table, as only these two showed Ct values.

To understand the impact of these numbers in the gene expression, samples were submitted to cDNA synthesis and evaluated in RT-qPCR test for β-actin expression.

### β-actin gene expression in the dura mater

**Figure 1:**
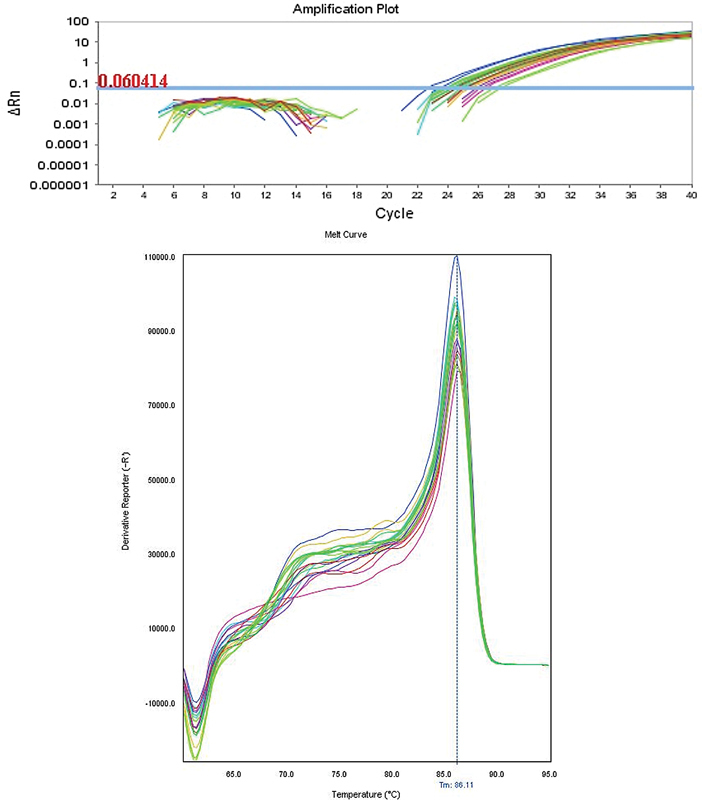
Dissociation curve at the end of the RT-qPCR.

The dissociation curve at the end of the RT-qPCR showed the peak temperatures of the curve in the range of 86.03°C to 86.22°C ( Figure 1 ). In the 1 ^st^ week of RT-qPCR, the best Ct average among duplicates, with a value of 22.4, was obtained using the RNA *later* /ReliaPrep method, followed by RNA *later* /RNeasy, with an average of 24.4. The TRIzol/ReliaPrep and TRIzol/RNeasy methods showed a small range of variation, with an average of 24.2 and 25.1, respectively. Regarding the 3-week evaluation, TRIzol/ReliaPrep showed the best values compared wth the RNA *later* /ReliaPrep method. The RNA *later* /RNeasy method, on the other hand, showed better results compared with the TRIzol/RNeasy method ( Table 1 ).

**Figure 2:**
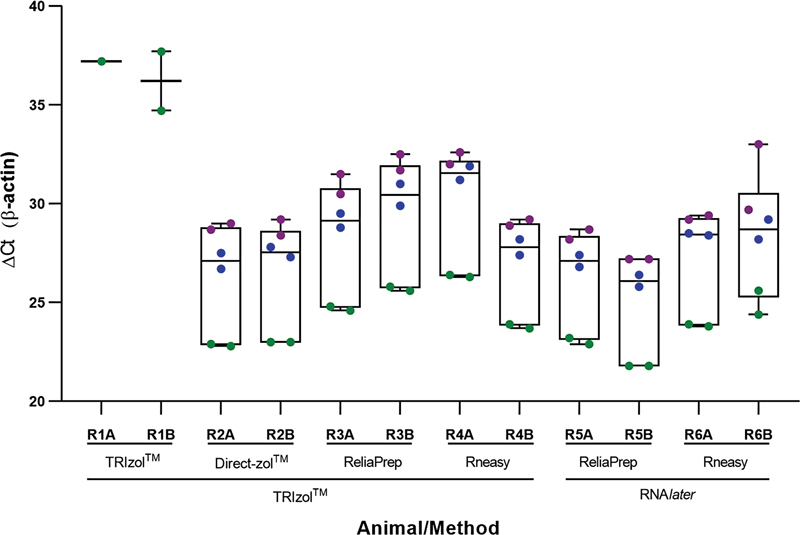
Intra-repeatability of the samples over three weeks.

The gene expression evaluation was performed three times, with an interval of one week between each evaluation. The TRIzol/TRIzol method showed no reproducibility in RT-qPCR. Despite the highest yield, this combination could not be associated with good gene expression results, since β-actin was undetectable in the 2 ^nd^ and 3 ^rd^ weeks of testing ( Figure 2 ). It is interesting to note that the methods based on TRIzol/Direct-zol and RNA *later* /ReliaPrep showed negative purity values, which suggest protein contamination, but the levels of gene expression were the best in both. In fact, the RNA *later* /ReliaPrep method showed the best Ct average over the 3 weeks ( Figure 2 ).

Lower Ct values were observed in the 1 ^st^ week for all samples, with a significant increment in the 2 ^nd^ and 3 ^rd^ weeks ( Figure 2 ). An intrarepeatability analysis demonstrated that the kits had similar Ct values between the duplicates, varying from 25.0 to 30.1 along the 3 weeks. In this interval, there was an increase of 4 cycles in Ct values compared with the 2 ^nd^ week, and 1 cycle for the 3 ^rd^ week of evaluation ( Figure 2 ). The mean and SD of the Ct values are shown in Table 1 . TRIzol/TRIzol has no evaluation since there were no Ct values throughout all analyzes.

Amplification was successful in the case of RNA *later* -based methods. However, for the TRIzol method, the use of a commercial kit based on affinity membrane method is suitable to assure higher yield and reproducibility in gene expression analysis.

## DISCUSSION

Traditionally, the levels of RNA gene expression in some tissues are low, making subsequent techniques difficult. 8 Although commercial kits include some specifications for different types of tissues in their protocols, the dura mater was not included in any of them, considering that it is not a tissue usually studied. The adaptations proposed for the dura mater RNA extraction protocols brought the addition of an incubation step before the beginning of the extraction, which implied the advancement of the cell lysis process. The RNA evaluation over 3 weeks allowed us to assess the quality and preservation of the material, in addition to the efficiency of each kit. These results were shown to be better in membrane-based protocols compared with purification with liquid-liquid kits.

There are several commercially available approaches for enriching RNA in biological samples, but most protocols result in very low yields due to a lack of specificity for the nature of the various existing tissues. Therefore, some protocol adaptations were found in the literature, but none for the dura mater. The dura mater has a strong relationship with neurovascular structures and, consequently, is an important tissue to evaluate the molecules that are released in the CNS, potentially involved in the pain mechanism. In addition, these optimizations do not include the evaluation of the methods of RNA stabilizing. 6 7 9 10 In order to determine the quality of a given sample, a spectrophotometer meets the demands with significant savings in time and money, short measurement cycle, and is easy to use, 11 although other methods can be used with higher precision, but also at a higher cost. 6 7 10

In the present study, the highest concentration values were obtained by the TRIzol method, but the RNA purity was poor. This occurrence was already described for adipose tissue processing, suggesting the chemical contamination of residues (proteins, traces of organic solvents and salts) as responsible for the overestimated concentration of nucleic acids. It also influences downstream analysis, leading to an increase in Ct and SD values (5,7), as observed in the present study for the dura mater. Thus, despite the low cost of the TRIzol method, it is important to note some inconveniences regarding harmful odors, laborious handling, in addition to the unsatisfactory purity that the method can present.

In contrast, the preservation in RNA *later* provides nucleic acids of high quality and conserved morphology for diverse tissues, besides not needing a quick-freezing system after immersing the sample. The method based on RNA *later* stabilization has a higher cost but does not emit toxic odors, as in the case of TRIzol, in addition to presenting yields as good as those of TRIzol, with a low rate of RNA contamination. 12 In addition, taking into account the data of purity, concentration, and Ct values, the samples stored in RNA *later* had the best results.

It is interesting to note that the samples with low levels of purity related to contamination by proteins (260/230), like TRIzol/Direct-zol and RNA *later* /ReliaPrep, showed the best Ct values for β-actin expression, which indicates that the evaluation of RNA yield and concentration could have been misled in the spectrophotometer evaluation.

The suggested adaptations may contribute to RNA extraction in human dura mater, since there are no structural differences between tissues. Furthermore, the study of gene expression of molecules present in the dura mater may contribute to elucidate mechanisms of diseases that are related to this structure, such as migraine. In addition, the physiopathogenic understanding of the behavior of structures and molecular units related to the dura mater aims to contribute to the development of tools and answers that clarify points in clinical research, and that these can contribute to application in possible therapeutic targets.

## LIMITATIONS OF THE STUDY

Although the adaptations suggested in the protocols are inexpensive, molecular biology techniques are generally very sensitive, requiring well-equipped laboratories and high-cost materials. Most of the data reported here are derived from dura mater samples; analyzing other similar tissues can expand the effectiveness of the method. Overcoming the challenges and limitations of RNA extraction protocols from specific tissues, such as the dura mater, will increase the use of molecular biology techniques and, thus, may contribute to the quality of analysis, providing better diagnostic tests.
